# Polo-like kinase 1 mediates BRCA1 phosphorylation and recruitment at DNA double-strand breaks

**DOI:** 10.18632/oncotarget.6825

**Published:** 2016-01-06

**Authors:** Corinne Chabalier-Taste, Laetitia Brichese, Carine Racca, Yvan Canitrot, Patrick Calsou, Florence Larminat

**Affiliations:** ^1^ Institute of Pharmacology and Structural Biology, CNRS UMR 5089, Toulouse, France; ^2^ University of Toulouse, UPS, Toulouse, France; ^3^ LBCMCP, Center for Integrative Biology, CNRS, Toulouse, France; ^4^ Equipe Labellisée Ligue Nationale contre le Cancer, Toulouse, France

**Keywords:** Plk1, DNA double-strand break, BRCA1, Rad51, homologous recombination, Chromosome Section

## Abstract

Accurate repair of DNA double-strand breaks (DSB) caused during DNA replication and by exogenous stresses is critical for the maintenance of genomic integrity. There is growing evidence that the Polo-like kinase 1 (Plk1) that plays a number of pivotal roles in cell proliferation can directly participate in regulation of DSB repair. In this study, we show that Plk1 regulates BRCA1, a key mediator protein required to efficiently repair DSB through homologous recombination (HR). Following induction of DSB, BRCA1 concentrates in distinctive large nuclear foci at damage sites where multiple DNA repair factors accumulate. First, we found that inhibition of Plk1 shortly before DNA damage sensitizes cells to ionizing radiation and reduces DSB repair by HR. Second, we provide evidence that BRCA1 foci formation induced by DSB is reduced when Plk1 is inhibited or depleted. Third, we identified BRCA1 as a novel Plk1 substrate and determined that Ser1164 is the major phosphorylation site for Plk1 *in vitro*. In cells, mutation of Plk1 sites on BRCA1 significantly delays BRCA1 foci formation following DSB, recapitulating the phenotype observed upon Plk1 inhibition. Our data then assign a key function to Plk1 in BRCA1 foci formation at DSB, emphasizing Plk1 importance in the HR repair of human cells.

## INTRODUCTION

The serine/threonine protein kinase Plk1 is the best-characterized member of the human Polo-like kinase family and is highly conserved from yeast to humans [1]. Plk1 contributes to a number of essential events throughout mitosis, such as CDK1-cyclin B activation, centrosome maturation, bipolar spindle formation and maintenance, chromosome segregation, spindle assembly checkpoint and cytokinesis [2]. It interacts with a number of proteins involved in these cell-cycle related events, but also with proteins implicated in translational control, RNA processing, replication licensing, DNA damage response and vehicle transport [3]. Plk1 typically binds CDK-phosphorylated targets through its Polo-box domains (PBD) and subsequently phosphorylates them [4]. It is expressed from early S to late M phases and its overexpression in many human tumors has been associated with tumorigenesis and poor prognosis [5]. Plk1 inhibitors are emerging as new anticancer agents in human tumors [5]. The cytotoxic effects of Plk1 inhibition alone are mainly due to the inhibition of the mitotic events in which it participates [5]. However, despite promising results of Plk1 inhibition *in vitro,* Plk1 inhibitors used as monotherapy only show partial antitumor activity in clinical trials [6].

In recent years, novel functions related to the DNA Damage Response (DDR) that orchestrates the appropriate repair of DNA double-strand breaks (DSB) have been described for Plk1 from S phase to mitosis. During mitosis, Plk1 blocks the Non-Homologous End-Joining (NHEJ) repair of DSB through inhibition of 53BP1 recruitment [7]. Under replication stress, Plk1 promotes the maintenance of pre-replicative complexes on dormant origins [8]. Plk1 has also been involved in the recovery of the G2 DNA damage dependent checkpoint [9] and in the phosphorylation of the Rad51 recombinase, an essential component of the homologous recombination (HR) DSB repair pathway [10]. HR uses an intact homologous sequence such as the undamaged sister-chromatid. It is essential for the resolution of stalled forks during DNA replication and participates also to the repair of exogenous damage such as these due to ionizing radiation (IR) [11]. This process is initiated by the binding of the MRN complex composed of Mre11, Rad50 and Nbs1 [12], followed by MRN- and CtIP-mediated resection to create 3′-overhanging ends that facilitates RPA loading and subsequent RPA-Rad51 exchange required for strand invasion [13]. Rad51-ssDNA nucleoprotein filament formation is highly regulated by several factors, such as the tumor suppressor BRCA1 [14]. BRCA1 facilitates CtIP-mediated DNA-end resection [15] and functions in a complex with BRCA2, PALB2 and Rad51 to promote the exchange of RPA by Rad51 [16]. Many proteins participating in the HR pathway form subnuclear foci through recruitment to, and accumulation at DNA damage sites [12]. These foci can be detected using immunostaining approaches and have become convenient markers for the presence of DSB (ATM-phosphorylated histone variant H2AX foci named γH2AX foci) or for the monitoring of HR (BRCA1 and Rad51 foci). BRCA1-deficient cells are not able to efficiently form Rad51 foci [17] and have impaired DSB repair by HR [18], resulting in genome instability and tumorigenesis [19].

Following exposure to IR, BRCA1 is phosphorylated at multiple sites by the checkpoint kinases ATM, ATR and Chk2 [20, 21]. Interestingly, it was shown that BRCA1 is also phosphorylated by CDK1 in response to IR and that this event is necessary for the efficient recruitment of BRCA1 to sites of DNA damage [22]. Based on the growing link between Plk1 and the HR repair pathway and on the fact that Plk1 targets CDK1-phosphorylated proteins, we hypothesized that Plk1 might regulate BRCA1 following DNA damage. In this study, we show for the first time that Plk1 inhibition impairs the ability of cells to repair DSB by HR and sensitizes cells to IR. Inhibition or down-regulation of Plk1 decreases BRCA1 foci formation following DNA damage. We further identified BRCA1 as a novel Plk1 substrate and determined that Ser1164 is the major phosphorylation site for Plk1 *in vitro*. In cells, mutation of Plk1 sites on BRCA1 significantly delays BRCA1 foci formation following DSB, recapitulating the phenotype observed upon Plk1 inhibition.

## RESULTS

### Inhibition of Plk1 before DNA damage sensitizes cells to IR and impairs DSB repair by HR

**Figure 1:**
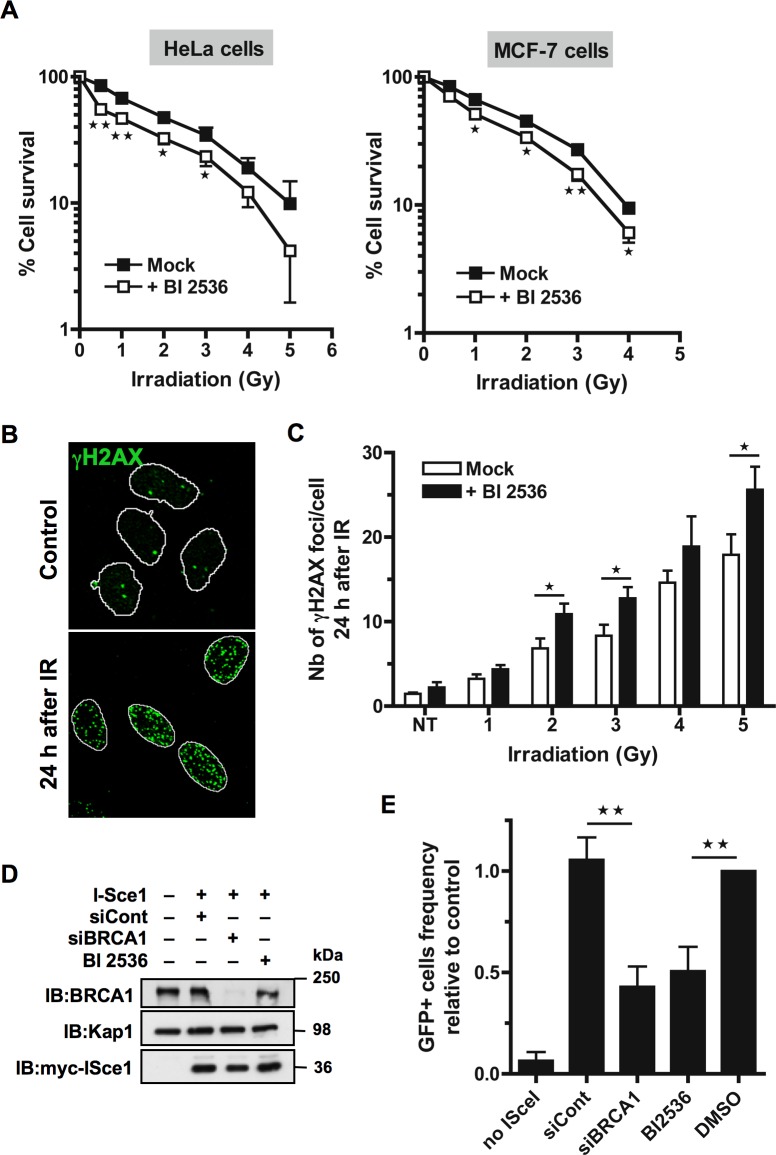
Inhibition of Plk1 sensitizes cells to ionizing radiations **A.** HeLa and MCF-7 cells are radio-sensitized when Plk1 is inhibited before IR. Cells were pre-treated or not with 10 nM BI2536 for 2 h before exposure to a range of doses of IR (0-5 Gy). Colonies were stained 10 to 12 days following IR and counted. Graph shows the mean percent surviving at each dose of IR relative to colonies formed at 0 Gy ± SE over 3 independent experiments done in triplicate. Significant differences in cell survival were assessed using a two-tailed paired Student's *t*-test and are indicated by * = *p* < 0.05; ** = *p* < 0.01.**B.** The incidence of γH2AX foci was determined in HeLa cells that were pre-treated or not with BI2536 for 2 h and then mock-exposed or exposed to IR, washed and collected 24 h after to perform immunofluorescence assay. Cells were immunostained with γH2AX antibody, probed with DAPI and then examined by confocal fluorescence microscopy. Representative images of γH2AX foci (green) in mock-exposed nuclei (control) or in irradiated nuclei 24 h following IR (4 Gy) are shown. **C.** The incidence of γH2AX foci is increased 24 h post-IR when Plk1 is inhibited. The number of foci was quantified using ImageJ software (NIH). Graph shows the mean number of γH2AX foci ± SE per cell over 3 independent experiments, *n* ≥ 100 cells per time-point. Significant differences in γH2AX foci number were assessed using a two-tailed unpaired Student's *t*-test and are indicated by * = *p* < 0.05. **D.** BRCA1 down-regulation by siRNA and expression of myc-tagged I-SceI endonuclease were analyzed after transfection with control siRNA (siCont) or siRNA against BRCA1 (siBRCA1) and subsequent transfection with myc-tagged I-SceI encoding plasmid (I-SceI) in presence or absence of Plk1 inhibitor (BI2536). Whole-cell extracts obtained from HEK293/DR-GFP cells were resolved by SDS-PAGE and immunoblotted using anti-BRCA1 and anti-myc antibodies. Equal loading was confirmed using anti-Kap1 antibody. **E.** DSB repair by HR is reduced in presence of Plk1 inhibitor. Frequencies of GFP-positive cells were measured by FACS following transient I-SceI expression for HEK293/DR-GFP cells treated with Plk1 inhibitor (BI2536) or mock-treated (DMSO), along with HEK293/DR-GFP cells transfected by siCont or siBRCA1. Graph shows the frequency of GFP-positive cells ± SE relative to the mean value of control DMSO-treated cells, n = 5. Significant differences in HR efficiency were assessed using a two-tailed paired Student's *t*-test and are indicated by ** = *p* < 0.01.

To investigate Plk1 function directly after DSB production during interphase, we used BI2536, a potent Plk1 inhibitor that instantly and reversibly inhibits Plk1 kinase activity [23]. We treated asynchronous cells with BI2536 for a short period before DNA damage, to ensure that the cells would be exposed to DSB with inhibited Plk1 activity but would not be arrested in mitosis at the time of DNA damage (mitotic index did not vary significantly following a short BI2536 treatment, data not shown). Failure to repair DSB causes sensitivity to IR, with NHEJ being important for survival following IR in all 3 stages of the interphase and HR primarily contributing to radioresistance in the late S and G2 phases [24]. We first examined whether Plk1 inhibition could sensitize cells to IR using a colony formation assay. HeLa and MCF-7 cells were treated with BI2536 or with DMSO vehicle for 2 h before exposure to IR. Following X-rays, cells were cultured for 10 to 12 days with a change of medium 24 h after IR to remove BI2536 and allow cell division. Our results showed that inhibition of Plk1 activity caused a significant reduction in the number of colonies using both cell lines (Figure 1A). Pre-treatment with BI2536 significantly reduced the radiation dose that causes a 50% decrease in the number of colonies (IC_50_) from 1.75 Gy to 1.05 Gy (*p*-value < 0.05) in HeLa cells and from 1.5 Gy to 1.1 Gy (*p*-value < 0.05) in MCF-7 cells (Figure 1A). These results indicate that pre-treatment with a Plk1 inhibitor sensitizes cells to IR, strongly suggesting that it might interfere with a DSB repair process.

Retention of IR-induced γH2AX foci is indicative of lethal DNA damage. To further assess the impact of Plk1 inhibition on DSB repair, we measured the incidence of residual γH2AX foci observed in cells 24 h after exposure to increasing IR doses. HeLa cells were treated with BI2536 or with DMSO vehicle for 2 h before IR and incubated for 24 h in presence or absence of BI2536, before γH2AX foci quantification. Representative images of untreated or irradiated cells used for the detection of γH2AX foci in nuclei are shown in Figure 1B. As expected, a higher frequency of γH2AX foci was found 24 h after DNA damage as the IR dose increased (Figure 1C). Compared to the mock-treated cells, BI2536 pre-treated cells exhibited significant higher numbers of residual γH2AX foci per irradiated cell 24 h following IR (*p*-value < 0.05; Figure 1C). The remaining number of γH2AX foci was approximately 20 to 30% higher in cells with inhibited Plk1 activity (Figure 1C), indicating that inhibition of Plk1 reduces the efficiency of DNA repair following IR in this cell line. Similar to what we observed in Hela cells, we detected more residual γH2AX foci 24 h following IR in MCF-7 cells that were pre-treated with BI2536 (*p*-value < 0.05; Supplementary Figure S1).

Finally, we directly assessed the impact of Plk1 inhibition on HR by using a well-established reporter system in which GFP expression indicates the occurrence of Rad51-dependent subtypes of homology-directed repair (HDR) events following induction of a unique DSB by transient expression of the rare-cutting I-SceI endonuclease [25]. Specifically, we used the cell line HEK293 that contains an integrated copy of the DR-GFP reporter consisting of a direct repeat of two inactive GFP alleles: a full length GFP interrupted by a recognition site for the I-SceI endonuclease and a second downstream modified GFP fragment that serves as a donor for HR-mediated repair of the DSB created by I-SceI cutting [26]. As a control for inhibition of I-SceI-induced HR, BRCA1 was depleted from the reporter cell line using siRNA (Figure 1D). Cells were subsequently transfected with an I-SceI expression vector [27], treated or not with BI2536 and the number of GFP-positive cells was assessed two days later by flow cytometry. Equivalent I-SceI expression levels were detected by immunoblot analysis for each independent transfection (Figure 1D). As described previously [18], HR directed repair was reduced in absence of BRCA1 compared to control siRNA transfection (*p*-value < 0.01; Figure 1E). Importantly, inhibition of Plk1 by BI2536 also significantly decreased the number of GFP positive-cells compared to DMSO-treated control cells (*p*-value < 0.01; Figure 1E). These data indicate that Plk1 activity is required for efficient DSB repair by HR. They are consistent with our cell survival analysis upon Plk1 inhibition since the extent of radio-sensitization is similar to what is observed in mutated BRCA1 cells that are HR-defective [28].

### Inhibition of Plk1 activity reduces BRCA1 foci formation following DNA damage

**Figure 2:**
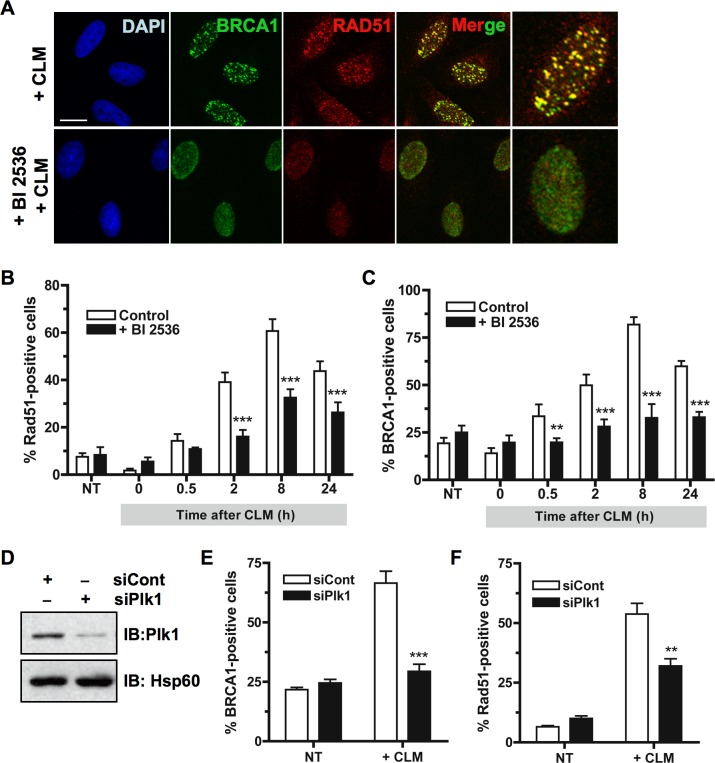
Inhibition of Plk1 impairs BRCA1 foci formation following DNA damage **A.** BRCA1 foci formation induced by DNA damaging treatment is impaired when Plk1 is inhibited. HeLa cells were pre-treated or not for 2 h with BI2536 and then treated with calicheamicin (CLM) for 1 h or left untreated, washed and collected at different time-points following treatment to perform immunofluorescence assay. Cells were immunostained with anti-BRCA1 and anti-Rad51 antibodies, probed with DAPI and then examined by confocal fluorescence microscopy. Representative images of BRCA1 (green) and Rad51 (red) co-staining in control cells (+ CLM) or in BI2536-pretreated cells (+ BI2536; + CLM) 2 h after CLM treatment are shown. **B.** The number of Rad51 foci-positive cells is reduced when Plk1 is inhibited. The number of foci was quantified using ImageJ software (NIH). Graph shows the mean number of positive cells containing more than 5 foci Rad51 foci ± SE over 3 independent experiments, *n* ≥ 120 cells per time-point. Significant differences in Rad51-positive cells numbers were assessed using a two-tailed unpaired Student's *t*-test and are indicated by *** = *p* < 0.001. **C.** The number of BRCA1 foci-positive cells is reduced when Plk1 is inhibited. The number of foci was quantified using ImageJ software (NIH). Graph shows the mean number of positive cells containing more than 5 foci BRCA1 foci ± SE over 3 independent experiments, *n* ≥ 150 cells per time-point. Significant differences in BRCA1-positive cells numbers were assessed using a two-tailed unpaired Student's *t*-test and are indicated by ** = *p* < 0.01 and *** = *p* < 0.001. **D.** Downregulation of Plk1 by siRNA was analyzed in HeLa cells. 24 h after transfection with 10 nM control siRNA (siCont) or siRNA against Plk1 (siPlk1), whole-cell extracts were resolved by SDS-PAGE and immunoblotted using anti-Plk1 antibody. Equal loading was confirmed using anti-Hsp60 antibody. **E.** The number of BRCA1 foci-positive cells is reduced when Plk1 is downregulated. BRCA1 foci were examined 6 h following CLM treatment. The number of foci was quantified using ImageJ software (NIH). Graph shows the mean number of positive cells containing more than 5 BRCA1 foci ± SE over 3 independent experiments, *n* ≥ 120 cells per time-point. Significant differences in BRCA1-positive cells numbers were assessed using a two-tailed unpaired Student's *t*-test and are indicated by *** = *p* < 0.001. **F.** The number of Rad51 foci-positive cells is reduced when Plk1 is downregulated. Rad51 foci were examined 6 h following CLM treatment. The number of foci was quantified using ImageJ software (NIH). Graph shows the mean number of positive cells containing more than 5 Rad51 foci ± SE over 3 independent experiments, *n* ≥ 120 cells per time-point. Significant differences in Rad51-positive cells numbers were assessed using a two-tailed unpaired Student's *t*-test and are indicated by ** = *p* < 0.01.

Plk1 was recently shown to phosphorylate Rad51 and to regulate damage-induced Rad51 localization [10]. We then examined the ability of endogenous Rad51 protein to form foci in cells that were treated with BI2536 before exposure to calicheamicin (CLM), a potent DSB inducer [29]. We chose CLM as DNA breaking molecule because it yields a much higher ratio of DSB to single-strand breaks *in cellulo*, compared to IR [30]. HeLa cells were treated with BI2536 or with DMSO vehicle for 2 h before CLM treatment for 1 hour and were then incubated for different time periods in presence or absence of BI2536. Rad51 foci were next visualized by immunofluorescence staining as shown in Figure 2A. Efficient formation of Rad51 foci was observed in control HeLa cells following CLM treatment (Figure 2A and 2B). However, when Plk1 was inhibited before DNA damage, the number of cells containing at least five detectable Rad51 foci (Rad51-positive cells) was significantly reduced over a 24 h time-course (*p*-value < 0.001; Figure 2B), consistent with the view that Plk1 is important for Rad51 recruitment at DSB sites [10]. We checked that pre-treatment with BI2536 before CLM treatment did not affect the extent of initial γH2AX signal (Supplementary Figure S2A).

In undamaged cells, BRCA1 forms discrete nuclear foci during S and G2 phases [31]. Following formation of DSB, these foci largely disappear and BRCA1 is recruited to damage sites to form larger foci colocalizing with γH2AX [32]. We next asked whether Plk1 activity acts upstream Rad51 recruitment and is necessary for the efficient recruitment of BRCA1 at DNA damage sites. HeLa cells were treated with BI2536 or with DMSO vehicle for 2 h before CLM treatment for 1 hour and were then incubated for different time periods in presence or absence of BI2536. As shown in Figure 2A, large BRCA1 foci were efficiently formed in control cells 2 h following CLM treatment. However, when cells were pre-treated with BI2536, BRCA1 foci were less efficiently formed and appeared smaller at the same time-point (Figure 2A). To accurately assess BRCA1 foci formation, we counted the number of cells containing at least five large BRCA1 foci (BRCA1-positive cells) over a 24 h time-course. Our data showed that the number of BRCA1-positive cells was significantly reduced when cells were pre-treated with BI2536 before DNA damage (*p*-value < 0.01; Figure 2C).

We confirmed these findings by examining BRCA1 foci formation following DSB when Plk1 was down-regulated using siRNA. Knockdown efficiency for Plk1 was analyzed by immunoblotting 24 h after transfection (Figure 2D). Quantification showed that siPlk1 reduced Plk1 expression to 15-20% of its endogenous level (Figure 2D). Down-regulation of Plk1 resulted in a significant reduction in both BRCA1 and Rad51 foci 6 h following CLM treatment (*p*-value < 0.01; Figure 2E and 2F). The extent of initial γH2AX signal induced by CLM was not affected by siRNA transfection targeting Plk1 (Supplementary Figure S2B). Altogether, our data indicate that Plk1 activity is necessary for efficient BRCA1 foci formation at sites of DSB.

### Plk1-BRCA1 complex formation is dependent on CDK1 activity

**Figure 3:**
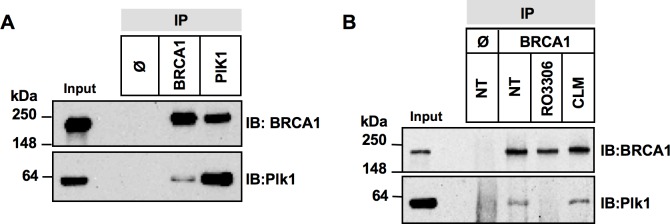
Complex formation between Plk1 and BRCA1 is reduced upon CDK1 inhibition **A.** BRCA1 and Plk1 co-immunoprecipitate *in cellulo*. Whole-cell extracts from asynchronous HeLa cells were incubated with anti-BRCA1 or -Plk1 antibody. Immune complexes were recovered with protein A-sepharose beads (IP) and analyzed by immunoblotting (IB) with anti-BRCA1 and anti-Plk1 antibodies. IP Φ, control IP. **B.** Complex formation between BRCA1 and Plk1 is reduced upon CDK1 inhibition. HeLa cells were left untreated (NT) or were treated with RO3306 for 4 h, or with calicheamicin (CLM) for 1 hour before co-immunoprecipitation. Equal quantities of whole-cell extracts were then incubated with anti-BRCA1 antibody. Immune complexes were recovered with protein A-sepharose beads (IP) and analyzed by immunoblotting (IB) with anti-BRCA1 and -Plk1 antibodies. IP Φ, control IP.

Since our data showed that Plk1 is necessary for efficient BRCA1 foci formation upon DNA damage, we next investigate whether BRCA1 can form a complex with Plk1. We assessed the co-immunoprecipitation of BRCA1 and Plk1 using antibodies against either protein in the cell lines used in this study. A constitutive complex formation was found in undamaged HeLa (Figure 3A) and MCF-7 cells (Supplementary Figure S3A), as previously observed in U2OS cells [33]. To bind Plk1, a substrate is usually primed by another kinase such as CDKs [34]. CDK1 has been shown to participate in DNA damage response pathways [35] and phosphorylates BRCA1 at S1497 and S1189/S1191 [36]. To test whether CDK1 inhibition alters Plk1-BRCA1 complex formation, we performed co-immunoprecipitation experiments following treatment with RO-3306, a specific CDK1 inhibitor [37]. Our results indicated that treatment by RO-3306 for 4 h strongly reduced BRCA1 phosphorylation at S1497, as demonstrated with a phospho-specific antibody (Supplementary Figure S3B) and abrogated Plk1-BRCA1 complex formation (Figure 3B). Interestingly we observed Plk1-BRCA1 co-immunoprecipitation immediately after DNA damage (Figure 3B). Taken together, our results indicate that Plk1 forms a complex with BRCA1 in a CDK1-dependent manner in cells under unperturbed conditions as well as conditions of genotoxic stress.

### BRCA1 is a substrate of PLK1

**Figure 4:**
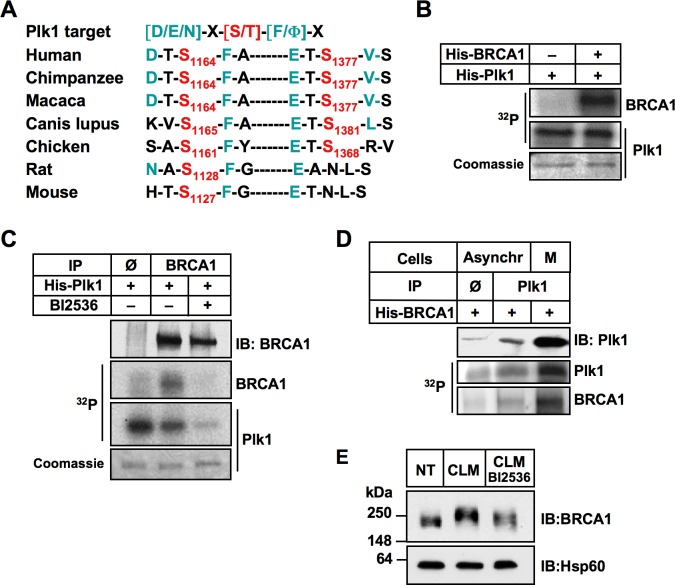
BRCA1 is a substrate of Plk1 **A.** Sequence alignment of orthologous BRCA1 regions. The two BRCA1 target sites containing a canonical target sequence for Plk1 phosphorylation are indicated. **B.** Recombinant His-BRCA1 protein is phosphorylated by recombinant active His-Plk1 protein in the presence of [γ^32^P]ATP. The reaction mixture was resolved by SDS-PAGE, followed by staining by Coomassie blue and autoradiography. Note that Plk1 phosphorylates itself. **C.** Endogenous BRCA1 is phosphorylated by recombinant active His-Plk1 protein in the presence of [γ^32^P]ATP. BRCA1 was immunoprecipitated from Hela cells and incubated with DMSO or with Plk1 inhibitor (BI2536) for 2 hours before addition of recombinant Plk1. The reaction mixture was resolved by SDS-PAGE, followed by staining by Coomassie blue and autoradiography. No signal was detected following BI2536 treatment, showing the specificity of the Plk1 kinase reaction. IP Φ, control IP. Note that Plk1 phosphorylates itself. **D.** Endogenous Plk1 phosphorylates recombinant His-BRCA1 protein in the presence of [γ^32^P]ATP. Plk1 was immunoprecipitated from asynchronous (lower Plk1 expression) or mitotic (higher Plk1 expression) cells and incubated with recombinant BRCA1. The reaction mixtures were resolved by SDS-PAGE, followed by autoradiography. IP Φ, control IP. **E.** Plk1 contributes to BRCA1 phosphorylation following DNA damaging treatment. Hela cells were left untreated (NT), or were treated with calicheamicin (CLM) for 1 hour in absence or presence of BI2536. Whole-cell extracts were resolved by SDS-PAGE and immunoblotted using anti-BRCA1 antibody.

We then examined whether BRCA1 was a Plk1 substrate. First, we used *in silico* analysis to predict putative kinase-specific phosphorylation sites in the BRCA1 sequence. Plk1 phosphorylates preferentially the [D/E/N]-X-[*S*/*T*]-[F/Φ]-X motif (with X, any amino acid; Φ, a hydrophobic amino acid) [38]. Interestingly, we found that S1164 and S1377 sites of BRCA1 are conformed to this consensus sequence, suggesting that Plk1 might interact with and phosphorylate BRCA1 (Figure 4A).

To investigate whether BRCA1 is directly phosphorylated by Plk1, we performed an *in vitro* kinase assay using human recombinant His-Plk1 and human recombinant His-BRCA1 as the substrate, in presence of [γ-^32^P] ATP. Kinase activity of purified His-Plk1 was confirmed using casein as substrate (Supplementary Figure S4A). As shown on Figure 4B, the ^32^P label was readily transferred to BRCA1 and also to Plk1 that autophosphorylates. We next analyzed endogenous BRCA1 phosphorylation by Plk1 using recombinant Plk1 protein and BRCA1 immunoprecipitated from HeLa whole-cell extracts (WCE) as a substrate. Our results showed that endogenous BRCA1 is indeed a substrate of Plk1 (Figure 4C). No BRCA1 phosphorylation was detected when BI2536 was added prior to the recombinant Plk1, indicating that the BRCA1 phosphorylation observed was Plk1-dependent (Figure 4C). Phosphorylation of endogenous BRCA1 by Plk1 was also detected using WCE obtained from MCF-7 cells (Supplementary Figure S4B).

The reciprocal *in vitro* kinase assay was performed using immunoprecipitated endogenous Plk1 and recombinant BRCA1 protein (Figure 4D). As expected, a much higher level of ^32^P incorporation was obtained onto recombinant BRCA1 using Plk1 immunoprecipitated from cells that were synchronized in mitosis using paclitaxel (PTX) (Figure 4D) since the maximal activity and expression of Plk1 coincide with the G2/M cell cycle phases [39]. Phosphorylation of BRCA1 following DNA damage results in a mobility shift in SDS-PAGE under adapted migration conditions [20, 21]. We next tested whether Plk1 inhibition may affect the phosphorylation of BRCA1 in response to DSB *in cellulo*. Cells were incubated 2 h in the presence or absence of BI2536 before treatment with CLM. WCE were analyzed by high resolution SDS-PAGE and immunoblotting with anti-BRCA1 antibody. Data revealed that inhibition of Plk1 slightly reduced the mobility of BRCA1 in condition of DNA damage treatment (Figure 4E). Taken together, our data demonstrate that BRCA1 is a novel substrate of Plk1.

### BRCA1 is phosphorylated by Plk1 mainly on Ser1164 *in vitro*

**Figure 5:**
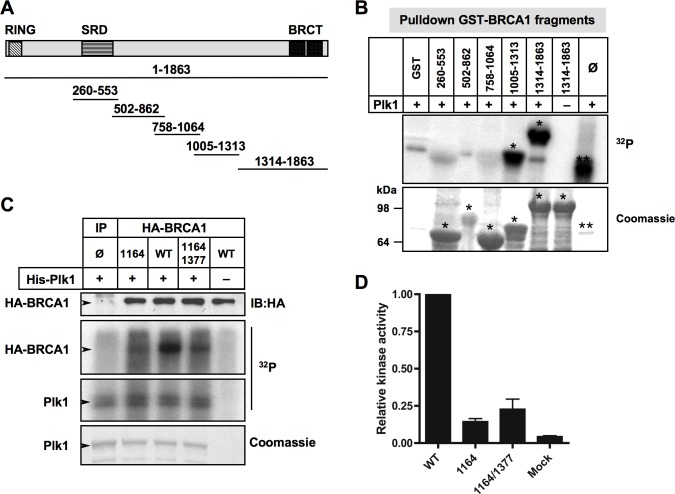
Plk1 phosphorylates BRCA1 mainly at Ser1164 residue *in vitro* **A.** Schematic illustration of GST-tagged BRCA1 fragments used as substrates in kinase reaction mixtures described in (B). **B.** Recombinant Plk1 protein phosphorylates GST-BRCA1 fragments aa1005-1313 and aa 1314-1863. His-Plk1 was incubated with purified GST-BRCA1 fragments and [γ^32^P]ATP to determine the Plk1-mediated phosphorylation. The asterisks in top panel (^32^P) mark GST-BRCA1 fragments that were phosphorylated with Plk1 (*) and Plk1 autophosphorylation (**). No signal was detected with GST alone or in absence of Plk1, indicating the specificity of the Plk1 kinase reaction. The SDS-PAGE gel stained by Coomassie blue illustrates the amount of purified GST-BRCA1 fragments used in the kinase reaction. The asterisks in bottom panel (Coomassie) indicate purified GST-BRCA1 fragments (*) and recombinant Plk1 (**). Φ, no addition of GST fragment. **C.** Recombinant Plk1 protein phosphorylates HA-tagged BRCA1 mainly on S1164. HeLa cells were transfected with plasmids encoding HA-tagged wild-type BRCA1 (WT), or mutated HA-tagged BRCA1 (S1164C, S1164C/S1377C). 24 h following transfection, HA-tagged BRCA1 proteins were immunoprecipitated and either subjected to immunoblotting or used in a kinase assay. Equivalent expression of the tagged proteins was confirmed by immunoblotting (IB) using anti-HA antibody. Immunoprecipitated HA-tagged BRCA1 proteins were incubated with recombinant Plk1 protein in the presence of [γ^32^P]ATP. The reaction mixtures were resolved by SDS-PAGE and visualized first by Coomassie blue staining and then with a FLA-3000 scanner. No [γ^32^P] signal was detected using control IP or without addition of Plk1 protein, showing the specificity of the Plk1 kinase reaction. Note that Plk1 phosphorylates itself. IP Φ, control IP. **D.** Histogram represents the relative phosphorylation of various immunoprecipitated HA-BRCA1 proteins by Plk1. [γ^32^P] signal detected via FLA-3000 Imager scans of the dried gels was quantified and presented as relative to level of [γ^32^P] incorporated in HA-wtBRCA1. Mock, control IP.

The two predicted consensus phosphorylation sites of Plk1 are located at sites Ser1164 (DTS^1164^FAE) and Ser1377 (ETS^1377^VSE) of BRCA1 sequence (Figure 4A). To show Plk1-dependent phosphorylation of BRCA1, we next performed an *in vitro* kinase reaction using purified GST-tagged BRCA1 fragments as substrates (Figure 5A). Equivalent levels of GST-tagged BRCA1 fragments were used as observed by Coomassie blue staining of the gel (Figure 5B, bottom panel), except for the GST-BRCA1 aa502-862 fragment that was found to be unstable, as shown previously [40]. As indicated in Figure 5B (upper panel), recombinant Plk1 strongly phosphorylates both BRCA1 fragments aa 1005-1313 and 1314-1863, in agreement with the location of the 2 putative phosphorylation sites that we identified *in silico*. To further confirm that these Ser residues are the Plk1 phosphorylation sites, we transfected plasmids encoding HA alone, HA-BRCA1wt, HA-BRCA1-S1164mut, or the double mutant HA-BRCA1-S1164/S1377mut into HeLa cells. The immunoprecipitated HA-BRCA1wt and HA-BRCA1 non-phosphorylatable mutants were subjected to a kinase assay with purified recombinant His-Plk1. Equivalent levels of immunoprecipitated wt or mutant proteins were used as Plk1 substrates (Figure 5C). As shown in Figure 5C, immunoprecipitated HA-wtBRCA1 was clearly phosphorylated by Plk1 and recombinant Plk1 underwent autophosphorylation. In contrast, mutations of S1164 and of both S1164 /S1377 significantly abrogated BRCA1 phosphorylation by Plk1 to nearly 20% of wild-type levels (Figure 5C and 5D). Interestingly, phosphorimager analysis of the kinase assays indicated that the S1164/S1377 double mutation did not decrease BRCA1 phosphorylation further than the S1164 single mutation (Figure 5D). Taken together our data indicate that Ser-1164 is most likely the major phosphorylation site for Plk1.

### Mutation of Plk1 sites on BRCA1 impairs BRCA1 foci formation following DSB

**Figure 6:**
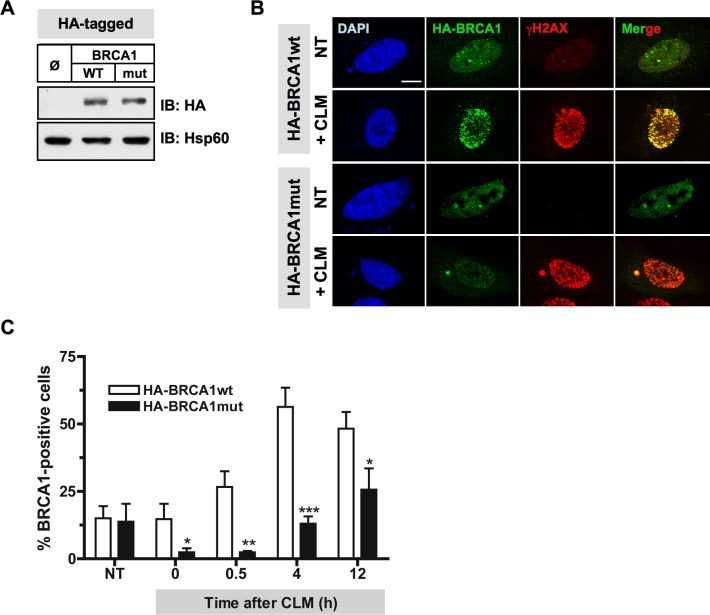
Mutations of Plk1 sites on BRCA1 compromise BRCA1 foci formation following DSB **A.** Immunoblotting using anti-HA and anti-Hsp60 antibodies indicates equivalent amounts of the HA-tagged BRCA1 proteins expressed in HeLa cells. **B.** BRCA1 foci formation induced by DNA damaging treatment is impaired when Plk1 sites are mutated. 24 h following transfection with HA-tagged BRCA1 constructs, HeLa cells were left untreated or treated with calicheamicin (CLM) for 1 h, washed and collected at different time-points following treatment to perform immunofluorescence assay. Cells were immunostained with anti-HA and anti-γH2AX antibodies, probed with DAPI and then examined by confocal fluorescence microscopy. Representative images of HA-BRCA1 (green) and γH2AX (red) co-staining in untreated cells (NT) or in CLM-treated cells 4 h after treatment are shown. **C.** The number of BRCA1-positive cells upon CLM treatment is reduced when BRCA1 is mutated at S1164/S1377. The number of foci was quantified using ImageJ software (NIH). Graph shows the mean number of positive cells containing more than 5 BRCA1 foci ± SE over 3 independent experiments, *n* ≥ 120 cells per time-point. Significant differences in BRCA1-positive cells numbers were assessed using a two-tailed unpaired Student's *t*-test and are indicated by * = *p* < 0.05, ** = *p* < 0.01 and *** = *p* < 0.001

To investigate the relevance of BRCA1 phosphorylation by Plk1 *in cellulo*, we then examined whether mutation of Plk1 sites identified on BRCA1 affects BRCA1 recruitment at DSB. HeLa cells were transfected with HA-tagged versions of the BRCA1 variants (Figure 6A). When HA-tagged wtBRCA1-transfected cells were treated with CLM for 1 h, characteristic HA-BRCA1 foci were formed following CLM washout and colocalized with the DSB marker γH2AX (Figure 6B). These findings indicate that exogenously expressed HA-wtBRCA1 was efficiently recruited to sites of DNA damage. However, when mutant HA-BRCA1 S1164/S1377 was expressed, a reduced number of cells containing BRCA1 foci was detected upon CLM treatment compared to HA-wtBRCA1 expression, although γH2AX signal induced by CLM was similar under both conditions (Figure 6B). The 12 h time-course of CLM-induced HA-BRCA1 foci containing HeLa cells showed that BRCA1 foci formation was significantly compromised at all time-points when BRCA1 was mutated on Ser1164/1377 residues (Figure 6B and 6C). We also noticed that the foci formed by the HA-BRCA1 mutant were often smaller than the ones detected with HA-wtBRCA1.

We confirmed these results using the BRCA1-mutated HCC1937 cells. HCC1937 cells that are derived from a homozygous BRCA1 deficient breast tumor, express a truncated and unstable form of BRCA1 [41] and are hypersensitive to DNA damaging agents [20]. HCC1937 cells were transfected with HA-tagged versions of BRCA1, treated with CLM for 1 h and then immunostained for the DSB marker γH2AX and for HA-BRCA1 foci. BRCA1 focus formation was less efficient at 12 h post-treatment when BRCA1 was mutated on S1164/S1377 residues (Supplementary Figure S5A), although γH2AX signal induced by CLM was similarly efficient (Supplementary Figure S5B). Altogether, our data indicate that mutations of Plk1 sites on BRCA1 significantly delay DSB-induced BRCA1 foci formation following DNA damage, recapitulating the phenotype observed upon Plk1 inhibition. They highlight the importance of Plk1-dependent phosphorylation in DSB-induced focus formation of BRCA1.

## DISCUSSION

Although Plk1 has been recently linked to DDR in mammalian cells [9, 10], its role(s) in this process remains to be clarified. The work described here provides new insights in the involvement of Plk1 in HR repair. We have shown that Plk1 activity is necessary for the efficient formation of BRCA1 foci following DSB and that Plk1 inhibition before DNA damage reduces DSB repair by HR and sensitizes cells to IR. Our data further indicate that Plk1 phosphorylates BRCA1 mainly on Ser1164 *in vitro*, a phosphorylation site that fits very well with the Plk1 target consensus sequence [38]. Importantly, mutation of Plk1 sites on BRCA1 impairs formation of BRCA1 foci after DNA damage, mimicking the phenotype observed with Plk1 inhibition or depletion and demonstrating that Plk1-mediated BRCA1 phosphorylation is necessary for optimal BRCA1 recruitment at DSB.

Plk1 is targeted to binding partners that have previously been ‘primed’ by phosphorylation [4]. Plk1 targeting requires two conserved polo-boxes (PBD) that act as a phosphopeptide-binding domain [42]. A large fraction of the known characterized PBD-dependent interactions involves a CDK1-dependent priming event [34]. Interestingly CDK1 phosphorylates BRCA1 at S1497 and Ser1189/1191 and its loss compromises BRCA1 foci formation following IR [36]. The data presented here show that the interaction between Plk1 and BRCA1 is dependent upon CDK1. It is then tempting to speculate that the CDK1 mediated-phosphorylation of BRCA1 creates a docking site for Plk1 and that this is the sequential phosphorylation of BRCA1 by CDK1 and Plk1 that allows optimal BRCA1 recruitment at damage sites.

BRCA1-containing complexes are crucial mediators of the DDR [43]. In particular, the BRCA1-PALB2-BRCA2 complex is essential for Rad51-mediated HR [44]. This complex functions to promote the exchange of RPA for Rad51, and contributes to sister-chromatid invasion. BRCA1 facilitates the recruitment of PALB2, BRCA2 and Rad51 to DSB sites and might further assist BRCA2 and Rad51 to promote strand invasion [44, 45]. It was recently shown that Plk1 phosphorylates Rad51 at Ser14, stimulating Rad51 recruitment to DNA damage [10]. The priming step is ensured by CDK-mediated phosphorylation of BRCA2 that triggers binding of Plk1, which in turn, phosphorylates Rad51 within the HR complex [46]. Our study revealed a new player in the molecular interplay between Plk1 kinase and the HR complex. We found that BRCA1 also is phosphorylated by Plk1 and that efficient BRCA1 recruitment at DSB is dependent on Plk1. In absence of Plk1 activity, BRCA1 accumulation into nuclear foci after DSB is compromised, leading to decreased Rad51 recruitment, HR repair defect and reduced cell survival to IR. Thus Plk1 has synergistic regulating effects on both BRCA1 and Rad51 proteins, providing combined means to attenuate or enhance HR-mediated repair of DSB.

Our data showed that Plk1 phosphorylates BRCA1 mainly on S1164 residue *in vitro*, in the middle of a canonical Plk1 target sequence. Interestingly, an extensive analysis of the phosphoproteome of HeLa cells has recently revealed the phosphorylation of Ser1164 of BRCA1 using mass spectrometry [47]. Moreover, a BRCA1 variant with a single nucleotide substitution (Ser1164Ile) has been detected in women with family history of breast and/or ovary cancer [48, 49]. This variant is predicted to be deleterious by Sorting Intolerant From Tolerant (SIFT; score > 0.05) [50] and possibly damaging by Polymorphism Phenotyping (Polyphen; score 0.602) [51]. Considering our current findings, abrogation of Plk1 phosphorylation site in this BRCA1 variant could very likely participate in the disruption of BRCA1 function in the tumor.

Plk1 inhibitors are emerging as potential anticancer agents, but their overall antitumor activity has been modest in trials performed so far [6]. Using Plk1 inhibitors in combination therapy may be an option to improve the clinical benefit of a conventional treatment, such as DNA damaging therapies. Although down-regulation of Plk1 through siRNA has been shown to radio-sensitize cells [52], Plk1 inhibition can cause radiosensitization or radioresistance depending on the treatment schedule [53]. Importantly, our data indicate that a treatment schedule in which a short Plk1 inhibition using BI2536 is performed before IR reduces the formation of BRCA1 and Rad51 foci, leading to impaired HR repair and to radiosensitization of cancer cells.

In conclusion, our data assign a key function to Plk1 in BRCA1 foci formation at DSB sites, emphasizing Plk1 importance in the HR repair of human cells.

## MATERIALS AND METHODS

### Cell culture and drug treatment

MCF-7, HeLa and HCC1937 cancer cells were obtained from the American Type Culture Collection (ATCC; Manassas, VA). Reporter cell line HEK293/DR-GFP was provided by Dr Jeremy Stark [54]. MCF-7 cells were grown in RPMI-1640 medium with L-Glutamine (Life Technologies) supplemented with 5% fetal bovine serum (Sigma-Aldrich), 50 μg/mL gentamycin and 1 μM insulin (Life Technologies). HeLa and HEK293/DR-GFP cells were grown in Dulbecco's modified Eagle's medium with L-glutamine (Life Technologies) supplemented with 10% fetal bovine serum (Lonza) and penicillin-streptomycin (Life Technologies). HCC1937 cells were grown in RPMI-1640 medium using ATCC recommendations. Where indicated, cells were treated with 50 pM calicheamicin-γ1 (CLM, a generous gift from PR Hamann, Wyeth Research, Pearl River, NY, USA) for 1 h, 50 nM paclitaxel (PTX, Sigma-Aldrich) for 16 h, 10 nM BI2536 (Plk1 inhibitor; Selleck Chemicals) for the indicated time periods or 10 μM RO-3306 (CDK1 inhibitor, Calbiochem) for 4 h.

### Clonogenic assay

Exponentially growing cells were plated into 6-wells plates at appropriate densities. The following day, they were incubated or not with BI2536 for 2 hours and then exposed at increasing doses of IR using an X-ray irradiator (Faxitron RX-650) or mock-exposed (0 Gy). After 24 h of incubation, cells were washed and incubated with fresh medium for further 10 to 12 days to allow colony formation. Plates were then washed with PBS and stained with 0.05% of crystal violet. Clusters containing > 50 cells were scored as colonies. All clonogenic assays were done 3 times in triplicate. The surviving fraction for a given dose was calculated as the ratio between the surviving irradiated colonies and the surviving mock-exposed colonies. The survival of the mock-exposed colonies was considered as 100%. GraphPad Prism software was use to fit curves to the mean surviving fraction and to calculate the IC_50_.

### Plasmid construction

Recombinant pcDNA_3_ plasmid encoding HA-tagged wtBRCA1 was described previously [21]. Mutant HA-BRCA1 plasmids were generated using the following mutagenic oligonucleotides: 5′-TGTTTTGCTGAAAATGA-3′ for the S1164C mutant and 5′-ATGTTTCACTCTCACACCC-3′ for the S1377C mutant. Mutagenesis was performed using the Multisite-directed Mutagenesis kit (Stratagene). Recombinant pcDNA_3_ vector encoding myc-tagged I-SceI endonuclease was reported previously [27].

### Transfection

Transient transfection of pcDNA_3_ vectors encoding HA-tagged wt BRCA1 or myc-tagged I-SceI was performed with Lipofectamine 2000 (Invitrogen) or with Fugene (Roche) using the manufacturer's recommendations. For control experiments, cells were transfected with empty pcDNA_3_-HA vector. Transfection of siRNA sequence pools directed against Plk1 or BRCA1 (On-Targetplus Smartpool^®^, Dharmacon) or control scrambled siRNA duplex (Dharmacon) was performed with 10 nM of the indicated siRNA duplexes using INTERFERin (PolyPlus Transfection).

### Immunoblot and immunoprecipitation analysis

Following transient transfection and/or drug treatment, whole-cell extracts (WCE) were prepared and immunoblots were performed as previously described [55]. The primary antibodies used to detect proteins were monoclonal anti-BRCA1 DO-9 (1: 200, Santa Cruz), monoclonal anti-HSP60 (1:1000, Sigma), monoclonal anti-HA (1:1000, Babco), polyclonal anti-pBRCA1Ser1497 (1:50, Upstate), polyclonal anti-γH2AX[pS139] (1:200, Cell Signaling Technology), monoclonal anti-Plk1 (1:500, Zymed), monoclonal anti-Myc epitope sequence (1:1000, Calbiochem) and polyclonal anti-Kap1 (1:2000; Abcam). The secondary antibodies were peroxidase-conjugated IgG (1:5000, Cell Signaling). Immunoblot signals were detected by using ECL (Pierce). For immunoprecipitation, 500 μg to 1 mg of precleared WCE were incubated with 10 μg of rabbit polyclonal anti-BRCA1 antibody (BD Biosciences) or 2 μg of rabbit polyclonal HA-probe Y-11 (Santa Cruz) or with 5 μg of rabbit polyclonal anti-Plk1 antibody (Calbiochem) at 4°C overnight. Immune complexes were recovered with protein A-sepharose beads (Pharmacia) and washed three times using lysis buffer. Then beads were subjected to Western Blot analysis. Control immunoprecipitation was performed without antibody.

### Recombination assay

10^5^ cells were seeded in 6-well plates and transfected with appropriate siRNA when necessary. 24 h later, cells were transfected with 1 μg of plasmid expressing I-SceI [27] and then treated with Plk1 inhibitor or mock-treated. 48 h after I-SceI transfection, cells were analyzed by flow cytometry (FACScalibur, Beckton-Dickinson) to quantify GFP-positive cells and measure HDR, as previously described [56]. Quantification was done on 50 000 sorted events. The background frequency of GFP+ cells was consistently less than 0.06%.

### GST-BRCA1 fragments production

Plasmids corresponding to GST-BRCA1 fragments were described previously and were constructed using the pGEX-5X3 vector (Pharmacia) and the human BRCA1 cDNA [40]. The GST-BRCA1 fusion proteins contain residues, 260 to 553, 502 to 802, 758 to 1064, 1005 to 1313, and 1314 to 1863 of BRCA1, respectively [40]. GST fusion proteins were expressed in *E. coli* BL21RP^+^ and purified as previously described [40]. Expression and purity of the different GST proteins were analyzed using SDS/PAGE followed by Coomassie blue staining.

### *In vitro* kinase assays

200 ng of recombinant active Plk1 kinase (Invitrogen) or 10 μl of Plk1 immunoprecipitated from asynchronous cells or from cells synchronized in mitosis with paclitaxel were used for the *in vitro* kinase assays. Plk1 was incubated with 0.3 μg of recombinant BRCA1 (Active Motif) or with 10 μl of BRCA1 immunoprecipated from asynchronous cells in Plk1 kinase buffer containing 20 mM Hepes pH 7.4, 50 mM KCl, 10 mM MgCl2 and 1 mM DTT. The reaction was started by the addition of 1 μl of 5 mM ATP (Pharmacia) and 1 μl of [γ^32^P] ATP (5 μCi) (Perkin-Elmer Life Sciences), incubated for 10-45 min at 30°C and stopped with sample buffer. Samples were separated by 7.5% SDS-PAGE gels and autoradiographed. All GST-tagged BRCA1 fragments were expressed in *E. coli* BL21 RP+. GST immunoprecipitates obtained following incubation of bacterial lysates with glutathione-Sepharose beads were washed with Plk1 kinase buffer. Equal amounts of each sample were next incubated with 20 μl of kinase buffer containing 5 μCi of [γ^32^P] ATP, 1 mM ATP and 200 ng of recombinant Plk1 for 30 min at 30°C and then separated by 10% SDS-PAGE. The gels were dried and autoradiographed. The kinase assay using HA immunoprecipitates obtained following HeLa transfection and immunoprecipitation using anti-HA antibody was performed as described above except for the extended reaction time (1 h). ^32^P activity was quantified using the Fuji Imager FLA-3000 after migration of proteins through 7.5% SDS-PAGE.

### Immunostaining

Cells were pre-extracted, fixed and permeabilized as previously described [57]. Incubation with relevant primary and secondary antibodies was carried out sequentially for 1 h each at room temperature. The primary antibodies used to detect proteins were monoclonal anti-BRCA1 DO-9 (1: 200, Santa Cruz), monoclonal anti-HA (1:1000, Babco), polyclonal Rad51 (1:200; Santa Cruz), and polyclonal anti-γH2AX[pS139] (1:100, Cell Signaling Technology). Secondary antibodies were Alexa Fluor^®^ 488 anti-mouse IgG (1:1000; Molecular Probes) and Alexa Fluor^®^ 594 anti-rabbit IgG (1:1000; Molecular Probes). DNA was stained using 0.05 μg/ml DAPI (Sigma-Aldrich) for 5 min. Slides were mounted with fluorescent mounting medium (Dako).

Images were captured using a confocal laser microscope (FV1000 Olympus; module TIRF with Hamamatsu OrcaR2 camera; Olympus Corporation) with a Plan-Apochromat 60xNA 1.40 oil immersion lens or a Plan-Apochromat 40X 0.95 lens and 405-, 488- and 559-nm lasers excitation. Images were taken with the same exposure time when comparing experimental conditions and were analyzed using Image J software (NIH).

### Statistical analysis

Results are presented as the mean ± SEM with significance calculated by two-tailed Student's t or Chi-square tests with standard software (GraphPad Prism, GraphPad Software, La Jolla, CA, USA). Significance was assigned for a *P*-value < 0.05. *, *P* < 0.05; **, *P* < 0.01; ***, *P* < 0.001.

## SUPPLEMENTARY MATERIAL FIGURES


