# Supplementary material for: Polo-like kinase 1 mediates BRCA1 phosphorylation and recruitment at DNA double-strand breaks
Source: Oncotarget. 2016 Jan 6;7(3):2269–83. doi: 10.18632/oncotarget.6825 (PMC4823034; doi:10.18632/oncotarget.6825)
Supplement: Supplementary file 1 [file oncotarget-07-2269-s001.pdf]

Polo-like kinase 1 mediates BRCA1 phosphorylation and recruitment at DNA double-strand breaks

Supplementary Material

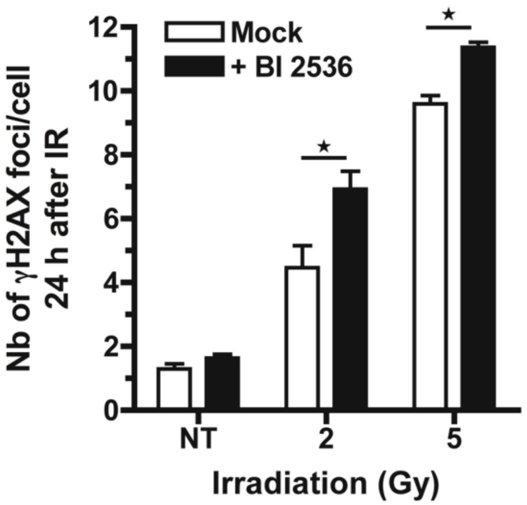

Supplementary Figure S1: Data related to Figure 1

Inhibition of Plk1 reduces the efficiency of DSB repair in MCF-7 cells.
The incidence of γH2AX foci was determined in MCF-7 cells that were pre-treated or not with BI2536 for 2 h and then mock-exposed or exposed to IR, washed and collected 24 h after to perform immunofluorescence assay. Cells were immunostained with γH2AX antibody, probed with DAPI and then examined by confocal microscopy. The number of foci was quantified using ImageJ software (NIH). Graph shows the mean number of γH2AX foci ± SE per cell over 3 independent experiments, n≥150 cells per time-point. *, $p<0.05$ (two-tailed unpaired Student's t test).

A

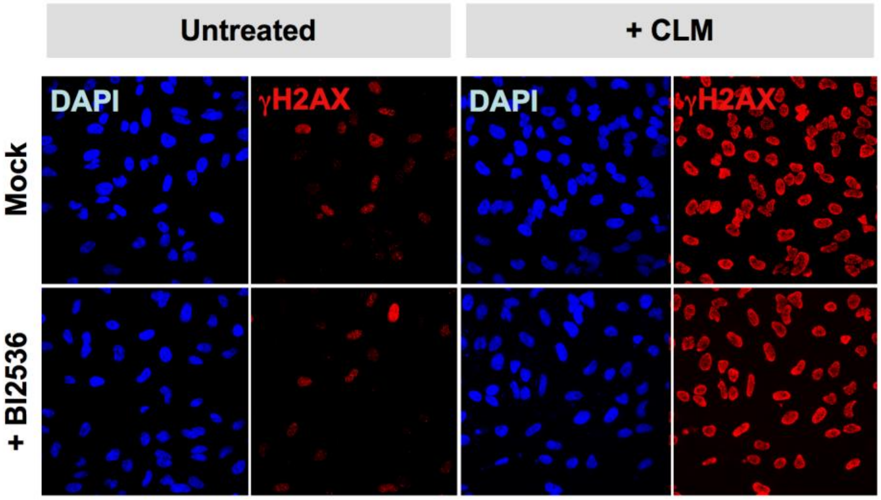

B

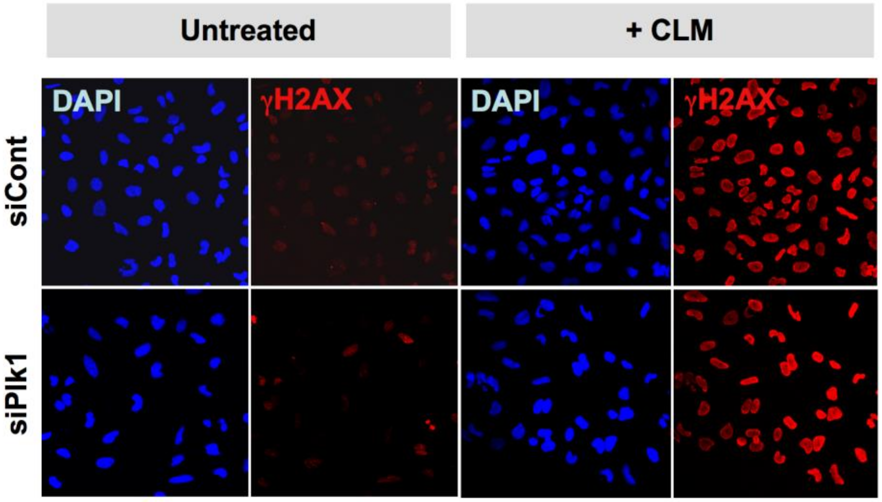

Supplementary Figure S2: Data related to Figure 2

Equivalent γH2AX signals are induced by CLM in absence of Plk1 activity.

A. Immunostaining using γH2AX antibody indicates equivalent amounts of CLM-induced DSB in cells pre-treated with BI2536 as compared to mock-treated cells. Cells were collected 30 min following CLM treatment to perform immunofluorescence assay.

B. Immunostaining using γH2AX antibody indicates equivalent amounts of CLM-induced DSB in cells pre-treated with siRNA against Plk1 as compared to control scrambled siRNA-treated cells. Cells were collected 30 min following CLM treatment to perform immunofluorescence assay.

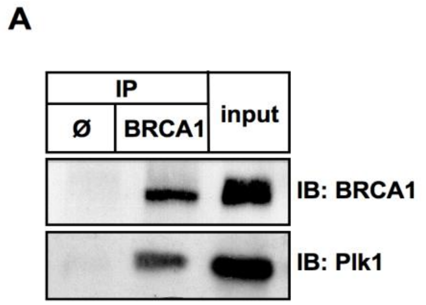

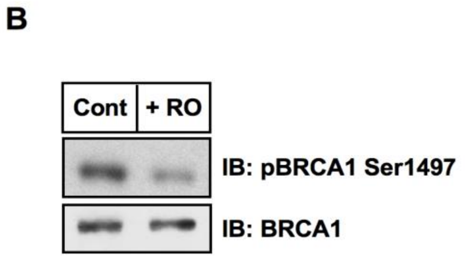

Supplementary Figure S3: Data related to Figure 3

Co-immunoprecipitation of endogenous BRCA1 and Plk1 is also observed in MCF-7 cells

A. Whole-cell extracts from asynchronous MCF-7 cells were incubated with anti-BRCA1 or -Plk1 antibody. Immune complexes were recovered with protein A-sepharose beads (IP) and analyzed by immunoblotting (IB) with anti-BRCA1 and anti-Plk1 antibodies. IP Φ, control IP.
B. Efficiency of pharmacological CDK1 inhibition using RO3306 (RO) is confirmed by reduced BRCA1 phosphorylation at Ser1497, a CDK1-phosphorylation site. Cells were incubated for 4 h with RO3306 before being harvested for western blot. Immunoblotting using pSer1497 BRCA1 antibody indicates that phosphorylation is reduced upon CDK1 inhibition.

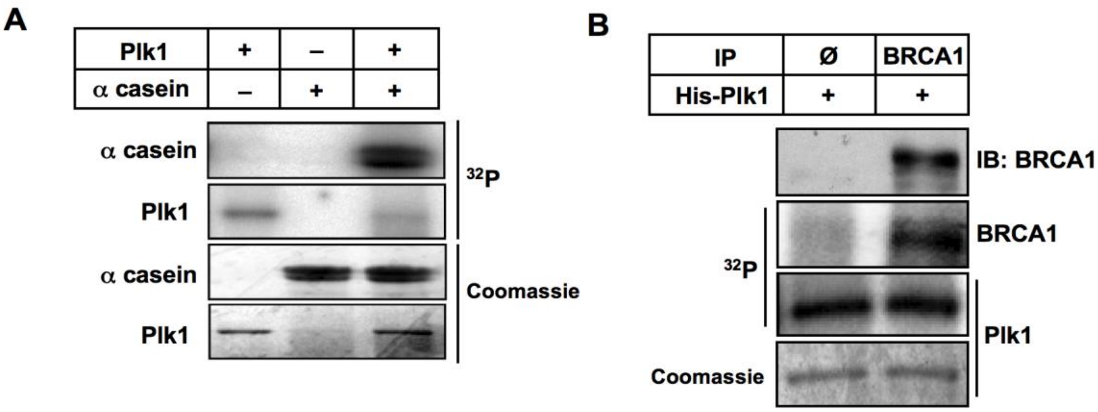

Supplementary Figure S4: Data related to Figure 4

Validation of the kinase assay with recombinant Plk1 using α-casein.

A. Kinase activity of recombinant His-Plk1 was assessed using α-casein, which is a common substrate for Plk1, in the presence of [γ^{32}P]ATP. The reaction mixture was resolved by SDS-PAGE, followed by staining by Coomassie blue and autoradiography. Note that Plk1 phosphorylates itself.

B. Endogenous BRCA1 is phosphorylated by recombinant His-Plk1 protein in the presence of [γ^{32}P] ATP. BRCA1 was immunoprecipitated from MCF-7 cells before phosphorylation by Plk1. The reaction mixture was resolved by SDS-PAGE, followed by staining by Coomassie blue and autoradiography. IP Φ, control IP.

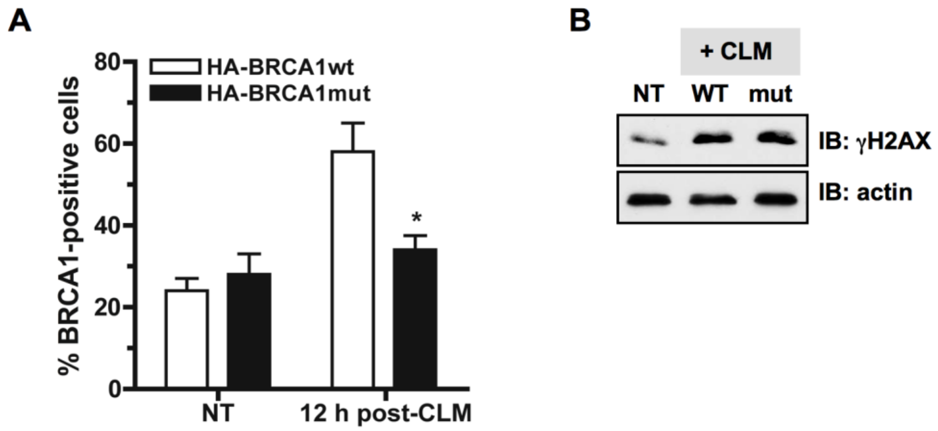

Supplementary Figure S5: Data relative to Figure 6

Mutations of Plk1 sites on BRCA1 reduce BRCA1 foci formation following DSB in BRCA1-deficient HCC1937 cells expressing HA-tagged BRCA1 proteins.

A. The number of BRCA1 foci-positive cells is reduced when BRCA1 is mutated at S1164/S1377. 24 h following transfection with HA-tagged BRCA1 constructs, HCC1937 cells were left untreated or treated with calicheamicin (CLM) for 1 h, washed and collected 12 h following treatment to perform immunofluorescence assay. Cells were immunostained with anti-HA antibody, probed with DAPI and then examined by confocal fluorescence microscopy. The number of foci was quantified using ImageJ software (NIH). Graph shows the mean number of positive cells containing more than 5 foci BRCA1 foci ± SE over 3 independent experiments, n≥150 cells per time-point. *, $p<0.05$ (two-tailed unpaired Student's t test).

B. Immunoblotting using γH2AX antibody indicates equivalent amounts of CLM-induced DSB in HCC1937 cells expressing HA-BRCA1wt or HA-BRCA1mut. Cells were collected 30 min following CLM treatment to perform Western blot. Equal loading was confirmed using anti-actin antibody.
